# Novel Engineered Carbon Cloth-Based Self-Cleaning Membrane for High-Efficiency Oil–Water Separation

**DOI:** 10.3390/nano13040624

**Published:** 2023-02-04

**Authors:** Nuo Chen, Kexin Sun, Huicong Liang, Bingyan Xu, Si Wu, Qi Zhang, Qiang Han, Jinghai Yang, Jihui Lang

**Affiliations:** 1 Key Laboratory of Functional Materials Physics and Chemistry of the Ministry of Education, Jilin Normal University, Siping 136000, China; chennuo0425@126.com (N.C.); skx19990501@126.com (K.S.); huicong0015@126.com (H.L.); xubingyan2022@163.com (B.X.); wusi9874@126.com (S.W.); qzh7512@126.com (Q.Z.); hanqiang@jlnu.edu.cn (Q.H.); jhyang1@jlnu.edu.cn (J.Y.); 2 Siping Hongzui University Science Park, Siping 136000, China

**Keywords:** CC-based membrane, doping and coating, oil–water separation, self-cleaning ability, oily wastewater treatment

## Abstract

A novel engineered carbon cloth (CC)-based self-cleaning membrane containing a Cu:TiO_2_ and Ag coating has been created via hydrothermal and light deposition methods. The engineered membrane with chrysanthemum morphology has superhydrophilic and underwater superoleophilic performance. The cooperativity strategy of Cu doping and Ag coating to the TiO_2_ is found to be critical for engineering the separation efficiency and self-cleaning skill of the CC-based membrane under visible light due to the modulated bandgap structure and surface plasmon resonance. The CC-based membrane has excellent oil–water separation performance when Cu is fixed at 2.5 wt% and the Ag coating reaches a certain amount of 0.003 mol/L AgNO_3_. The contact angle of underwater oil and the separation efficiency are 156° and 99.76%, respectively. Furthermore, the membrane has such an outstanding self-cleaning ability that the above performance can be nearly completely restored after 30 min of visible light irradiation, and the separation efficiency can still reach 99.65% after 100 cycles. Notably, the membrane with exceptional wear resistance and durability can work in various oil–water mixtures and harsh environments, indicating its potential as a new platform of the industrial-level available membrane in dealing with oily wastewater.

## 1. Introduction

The oil–water mixture as a pollutant with wide sources causes a lot of economic losses and induces a huge hazard to our living environment [1,2,3,4,5,6]. Some important procedures including gravity separation, coagulation, air flotation and demulsification have been provided for separating oily wastewater [7,8]. These methods, however, have some drawbacks such as low separation efficiency, secondary pollution and high cost [9,10,11]. Therefore, the practical work for solving the oily wastewater problem is an essential and urgent challenge [12,13,14]. Superwettability materials, particularly superhydrophilic and underwater superoleophobic materials, have become a popular topic in recent years [15]. This kind of material with low surface energy usually has high separation efficiency. However, due to oil adhesion from constant use, they are readily polluted and obstructed by oil droplets [16,17,18,19,20,21]. Hence, the investigation of materials with excellent self-cleaning ability has been undertaken, and some materials including CC, metal mesh, filter membrane, etc., have been proposed [22]. Because of its higher flexibility, good mechanical properties and long-term viability, the CC membrane is an excellent choice for oil–water separation [23,24,25,26]. As an excellent catalyst material, TiO_2_ has excellent catalytic performance under ultraviolet (UV) light irradiation and can realize self-cleaning pollutants on the membrane surface during the process of oil–water separation [27,28,29]. However, its application is limited because it only responds to ultraviolet light (5% of sunlight) [30,31]. Therefore, it has great significance to developing its self-cleaning performance with visible light responsiveness. The strategies of doping and coating are both effective techniques for modifying the optical band gap of the materials in the visible region and can even enhance catalytic performance [32,33,34]. The transition metal of d-states Cu doping can generate intermediate energy states between the host material’s valence and conduction bands, preventing the recombination of electron–hole (e^−^/h^+^) pairs effectively [35,36]. The stable emission in the visible region may be obtained by Cu doping as well as in the near-IR region for various optical materials. When Ag is encapsulated into the TiO_2_ system, either Ag clusters or their cations can serve as active sites in catalytic reactions to construct highly efficient catalysts because of its special effect on the separation of photogenerated e^−^/h^+^ when exposed to visible light [37,38,39,40]. Additionally, the deposition of precious metals shows obvious plasma resonance and broadens the absorption range of visible light. Therefore, it is believed that the cooperativity strategy of Cu doping and Ag coating in the CC@TiO_2_ system may induce the desired performance in oil–water separation application.

According to the information presented above, a novel engineered CC-based self-cleaning membrane for highly efficient oil–water separation is constructed by assembling Cu:TiO_2_ and Ag coating to modify the CC via hydrothermal and light deposition methods. The synergistic design for the engineered membrane can possess an outstanding oil–water separation capability; additionally, it can realize an excellent self-cleaning ability under visible light irradiation. For practical applications in industry, the harsh environment test of the engineered membrane is also analyzed in depth.

## 2. Experimental

### 2.1. Materials and Synthesis

The used CC was created by annealing the cotton fiber supplied by Xitaotao Trading Co., Ltd. Tetrabutyl titanate (C_16_H_36_O_4_Ti), silver nitrate (AgNO_3_), copper nitrate trihydrate (Cu(NO_3_)_2_·3H_2_O), Sudan III (C_22_H_16_N_4_O), methylene blue (C_16_H_18_ClN_3_S·3H_2_), 1, 2-dichloroethane (C_2_H_4_Cl_2_) and n-hexane (C_8_H_14_) were purchased from Sinopharm Group Chemical Co., Ltd. Beijing Chemical Plant supplied the hydrochloric acid (HCl) and toluene (C_6_H_5_CH_3_). For the oil–water test, various oils were prepared to form the oil–water mixtures. Aladdin Industrial Corporation provided petroleum ether (boiling point 90~120 °C). Soybean oil was bought at a nearby supermarket. The experiment employed deionized water as the experimental water, and all chemical reagents were of an analytical degree.

The preparation process of the engineered CC-based membranes was shown as follows. First, the cotton fibers were divided into the same size of 3 cm × 3 cm and washed 3~5 times with ethanol and deionized water, and then the cleaned cotton fiber cloth was dried in an oven at 80 °C for several hours. Second, the dried cotton fiber cloth was carbonized in a nitrogen atmosphere at 500 °C for 2 h, heated at a rate of 5 °C/min. The CC was produced after carbonization and rinsed three times with deionized water and 6 wt% hydrochloric acid, respectively. The washed CC was then dried in a drying oven at 80 °C for 2 h. Third, the cleaned CC was soaked in alcohol for 10 min for further use. Fourth, 20 mL of concentrated hydrochloric acid, 20 mL of deionized water, 2.5% Cu(NO_3_)_2_·3H_2_O (Cu:Ti = 2.5 wt%, the ratio that was the best in our previous report) and 2.5 mL of tetrabutyl titanate were mixed, and then a light-yellow mixed solution was formed. Following that, the hydrothermal reaction was carried out and the above solution as well as the prepared CC were removed into a PTFE reactor at 150 °C for 5 h. After cooling the PTFE reactor to an ambient temperature, the CC-based membrane containing Cu:TiO_2_ was cleaned and then dried in an oven at 80 °C. Finally, the above membrane was soaked in 30 mL of 0.001 mol/L, 0.002 mol/L, 0.003 mol/L, 0.004 mol/L, 0.005 mol/L and 0.006 mol/L AgNO_3_ under ultraviolet light. After cleaning and drying, the novel engineered CC-based membranes with different Ag contents were synthesized. For convenience, these membranes were named CC@Cu:TiO_2_@Ag_Y_ (Y = 0.1, 0.2, 0.3, 0.4, 0.5 and 0.6).

### 2.2. Characterizations

A contact angle instrument was used to measure the contact angle (CA) of the CC@Cu:TiO_2_@Ag_Y_ membranes (JC2000CD, AC 220 V and 50 Hz). The membrane structure was determined using an X-ray diffractometer (XRD, Rigaku-D/max-2500, Tokyo, Japan). The morphology, valence and composition state of the membranes were observed by a scanning electron microscope (SEM, JSM-7800F, Osaka, Japan), an energy dispersive spectrometer (EDS, 51-XMX1112, London, UK) and an X-ray photoelectron spectrometer (XPS, VGESCALAB 250×, Mark II, London, UK).

## 3. Results and Discussion

**Figure 1:**
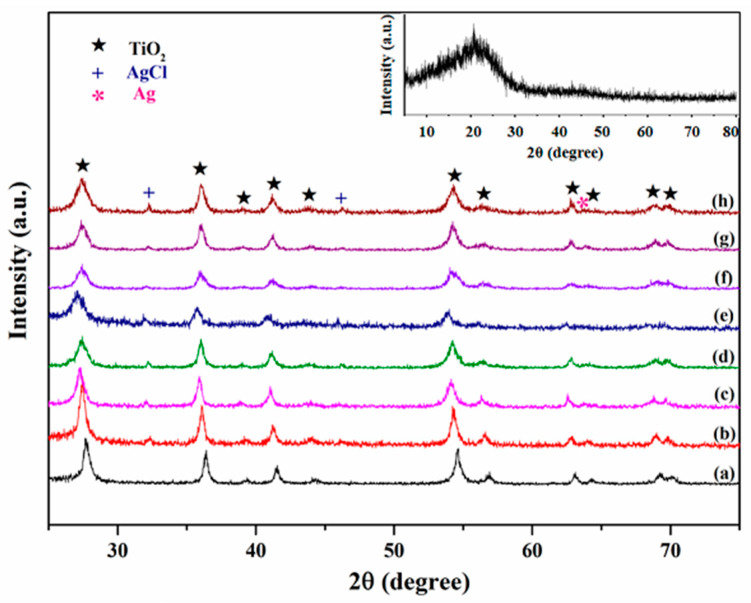
XRD patterns of (**a**) CC@TiO_2_, (**b**) CC@Cu:TiO_2_ and (**c**–**h**) CC@Cu:TiO_2_ @Ag_Y_ (Y = 0.1, 0.2, 0.3, 0.4, 0.5 and 0.6) membranes, and the inset shows the XRD pattern of CC substrate.

The XRD patterns of the CC, CC@TiO_2_, CC@Cu:TiO_2_ and CC@Cu:TiO_2_@Ag_Y_ (Y = 0.1, 0.2, 0.3, 0.4, 0.5 and 0.6) membranes are shown in Figure 1. Compared to the original CC in the inset, some strong diffraction peaks appear in the patterns of Figure 1a–h. For CC@TiO_2_ in Figure 1a, the peaks are located at 27.76°, 36.43°, 39.26°, 41.53°, 44.26°, 54.64°, 56.87°, 62.12°, 64.43°, 69.23° and 70.13°, which correspond to the diffraction of rutile TiO_2_ crystal planes (110), (101), (200), (111), (210), (211), (220), (002), (310), (301) and (112) [41,42,43]. It means that the rutile TiO_2_ is effectively formed on the CC. Compared to the CC@TiO_2_, the Cu-doped membranes in Figure 1 lack any diffraction peaks for Cu ions (b-h), indicating that Cu ions may be doped into the TiO_2_ matrix. Moreover, the position of the main diffraction peaks of the Cu-doped membranes has a slight shift to a smaller angle compared with the CC@TiO_2_ in Figure 1a, illustrating that the lattice parameters of the Cu-doped membranes are a little larger than those of the undoped ones. By fitting the XRD data with the least squares method, the lattice parameters of the two membranes for CC@TiO_2_ and CC@Cu:TiO_2_ are calculated. The calculated results of the former (a = 4.593 nm, c = 2.959 nm) and the latter (a = 4.613 nm, c = 2.963 nm) indicate that Cu ions have been incorporated into the TiO_2_ lattice and may have replaced the Ti ion sites due to the larger ionic radius of Cu ions (R(Cu^+^) = 0.096 nm and R(Cu^2+^) = 0.072 nm) [44,45,46,47,48]. For the CC@Cu:TiO_2_@Ag_Y_ membranes shown in Figure 1c–h, the peaks at 32.24° and 46.27° belong to AgCl, and the peak at 63.83° belongs to Ag [49]. In addition, it is found that the intensity of the peak related to the Ag element is enhanced gradually with the increase in Ag content, indicating the successful coating of the Ag element on the surface of the CC@Cu:TiO_2_ membrane.

**Figure 2:**
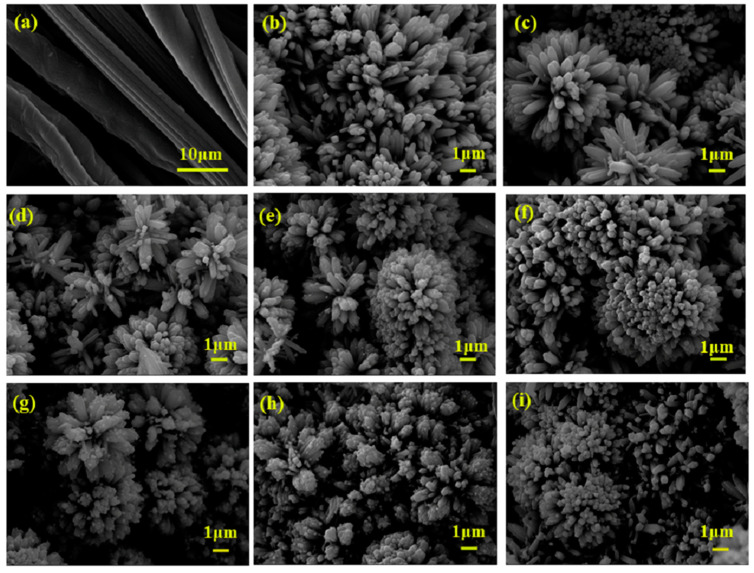
SEM images of (**a**) CC, (**b**) CC@TiO_2_, (**c**) CC@Cu:TiO_2_ and (**d**–**i**) CC@Cu:TiO_2_@Ag_Y_ (Y = 0.1, 0.2, 0.3, 0.4, 0.5 and 0.6) membranes.

The results of the SEM test are shown in Figure 2 to investigate the morphology of the membranes. In Figure 2a, the original CC is composed of fibers interlaced with each other to form the 3D network structure. When the TiO_2_ nanomaterials grow on the CC, the original CC surface cannot be seen on the CC@TiO_2_, indicating that the CC is fully covered by numerous TiO_2_ nanorods and that these nanorods with diameters of 150~200 nm are arranged closely. Because the original CC fibers are not the planar construction, the nanorods on the CC grow in a flower morphology (the CC@TiO_2_ membrane in Figure 2b). After Cu doping, it is found that the flower structure is more evident, and a chrysanthemum-like structure on the original basis is formed for the CC@Cu:TiO_2_ as a result of the Cu-O-Ti in the crystal lattice shown in Figure 2c. The Cu-O-Ti groups hindering the crystal growth are formed during the initial growth process, and then some Ti ion sites might be replaced by the Cu ions to evoke the interfacial energy of the nanorod seeds, which results in the nanorods becoming denser on the flower structure. Therefore, the chrysanthemum-like structure of the membrane is eventually formed with Cu doping in our case [50]. The morphology of the CC@Cu:TiO_2_@Ag_Y_ membranes has been slightly modified for additional Ag coating. It is found that some Ag particles with diameters of 200~250 nm are attached to the chrysanthemum surface, and the amount of Ag particles are gradually increased by increasing the Ag content shown in Figure 2d–i.

**Figure 3:**
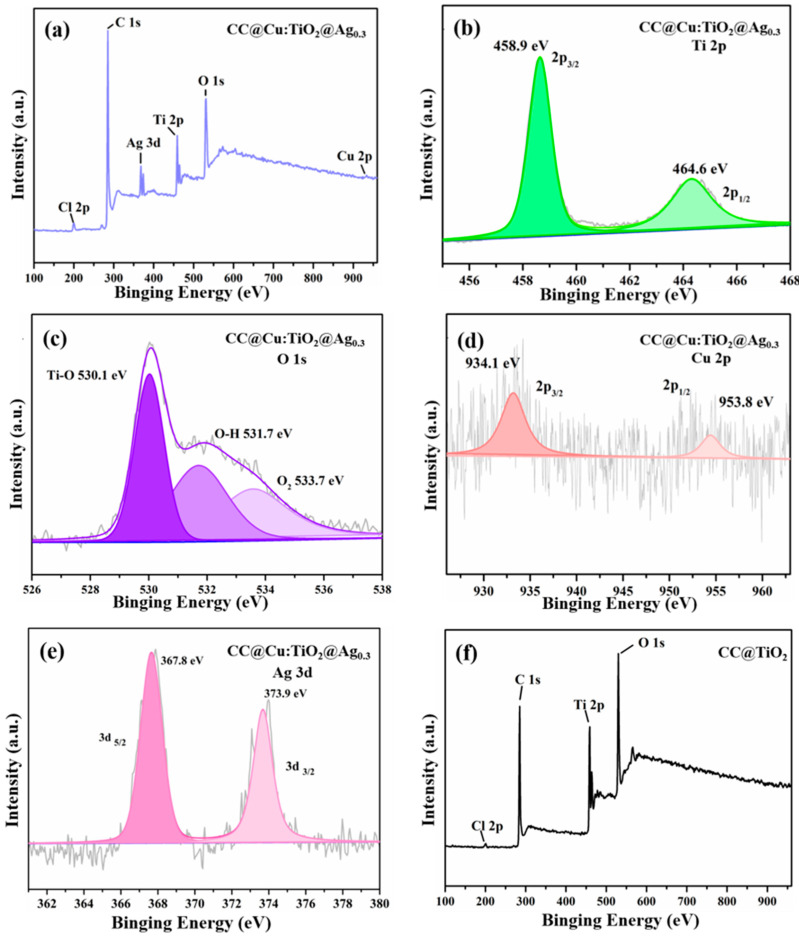
XPS spectra of (**a**–**e**) CC@Cu:TiO_2_@Ag_0.3_ and (**f**) CC@Cu:TiO_2_ membranes.

**Figure 4:**
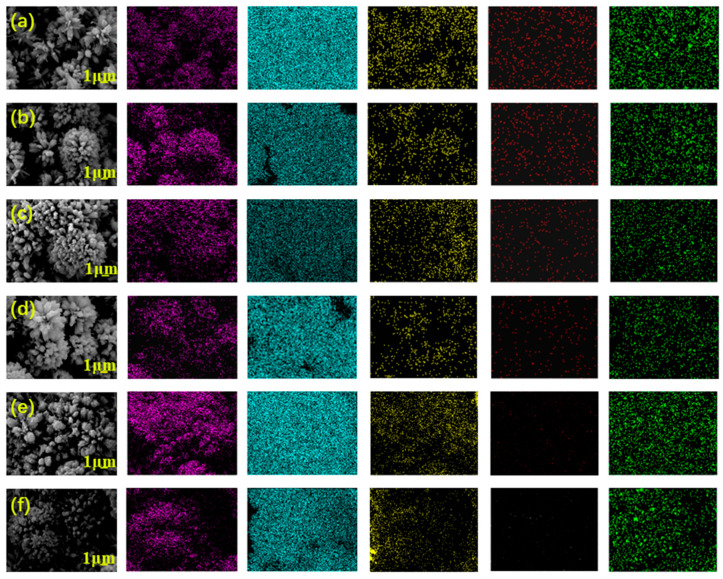
SEM images and the corresponding EDS mappings of (**a**) CC@Cu:TiO_2_@Ag_0.1_, (**b**) CC@Cu:TiO_2_@Ag_0.2_, (**c**) CC@Cu:TiO_2_@Ag_0.3_, (**d**) CC@Cu:TiO_2_@Ag_0.4_, (**e**) CC@Cu:TiO_2_@Ag_0.5_, (**f**) CC@Cu:TiO_2_@Ag_0.6_ membranes.

In Figure 3, the XPS spectrum of the CC@Cu:TiO_2_@Ag_0.3_ membrane is used to investigate the composition and chemical state of these elements. In the CC@Cu:TiO_2_@Ag membrane, six elements including Ti, O, C, Cl, Cu and Ag are shown in Figure 3a. The C element comes from the original CC, the Ti and O elements come from TiO_2_, and Cu(NO_3_)_3_H_2_O and AgNO_3_ are the sources of the Cu and Ag elements. In Figure 3b of the Ti 2p spectrum, the peak of Ti 2p_3/2_ is located at 458.9 eV and Ti 2p_1/2_ is located at 464.6 eV, which is consistent with the position of Ti^4+^ in the oxide [51]. In Figure 3c of O1s, the peak of O1s can be fitted to O_Ti-O_ at 530.1 eV, O_O-H_ at 532.1 eV and adsorbed O_2_ at 533.7 eV [52]. The Cu 2p has two peaks at 934.1 eV (Cu 2p_3/2_) and 953.8 eV (Cu 2p_1/2_) in Figure 3d [53], which confirms the existence of Cu^2+^ ions. The peak of Ag 3d in Figure 3e can be fitted to two peaks at 367.8 eV (Ag 3d_5/2_) and 373.9 eV (Ag 3d_3/2_), respectively, and these values indicate that Ag exists in the form of Ag^0^ and Ag^+^ in the CC@Cu:TiO_2_@Ag_0.3_ membrane [54,55]. Based on the presence of Cl^−^ in the membrane from the XPS and XRD results, it is assumed that Ag and AgCl are formed from the same Ag source. In our case, the Cl^−^ is thought to be associated with the CC. To demonstrate this, the XPS spectrum of a CC@TiO_2_ membrane is shown in Figure 3f for comparison. From this spectrum, the four elements of Ti, O, C and Cl appear in the membrane, and it confirms that the Cl^−^ is from the synthesized process of the CC. For researching the distribution of these elements, the EDS mappings of the CC@Cu:TiO_2_@Ag_Y_ (Y = 0.1, 0.2, 0.3, 0.4, 0.5 and 0.6) membranes are given in Figure 4. From the mappings, it is found that the elements, especially the Cu and Ag, are all uniformly distributed in these membranes, indicating the uniformity of Cu doping and Ag coating. When these membranes with different Ag contents are compared, it is clear that the Ag content increases as the AgNO_3_ content increases. It shows that the Ag coating on the membranes gradually increases, which corresponds to the XRD results.

**Figure 5:**
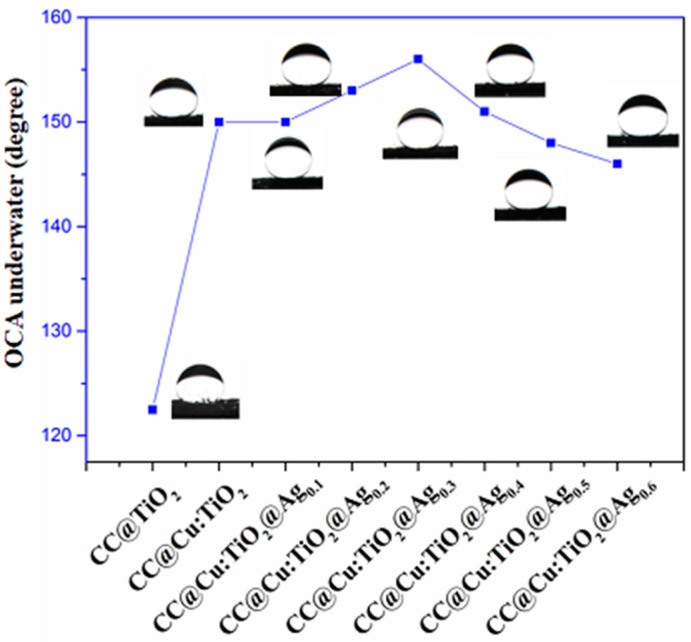
The U-OCA of CC@TiO_2_, CC@Cu:TiO_2_ and CC@Cu:TiO_2_@Ag_Y_ (Y = 0.1, 0.2, 0.3, 0.4, 0.5 and 0.6) membranes.

**Figure 6:**
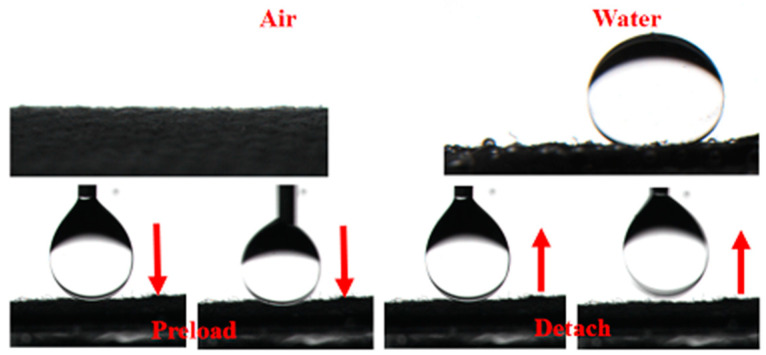
The hydrophilicity of CC@Cu:TiO_2_@Ag_0.3_ membrane in the air, the oleophobicity of CC@Cu:TiO_2_@Ag_0.3_ membrane in the water and its dynamic oleophobic diagram.

The underwater oil contact angle determines the membrane’s wettability (U-OCA). Compared to the original CC, the wettability of the CC@TiO_2_, CC@Cu: TiO_2_ and CC@Cu:TiO_2_@Ag_Y_ (Y = 0.1, 0.2, 0.3, 0.4, 0.5 and 0.6) membranes changes significantly. The U-OCA of CC and the CC@TiO_2_ membranes are 0° and 122.5°, shown in Figure 5, respectively. When doping Cu ions into a TiO_2_ host, the U-OCA of the CC@Cu:TiO_2_ increases to 150°. In addition, the U-OCA of the CC@Cu:TiO_2_@Ag_Y_ also varies with increasing the Ag content from 0.1 to 0.6, and these values are 150°, 153°, 156°, 151°, 148° and 146°, respectively. Among them, the CC@Cu:TiO_2_@Ag_0.3_ membrane has the largest U-OCA. Cu doping and Ag coating are thought to take part in promoting the U-OCAs of the membranes. On the one hand, when the membrane is wet, a hydration layer forms on the outside of the membrane due to capillary tension. The water molecules adsorbed on the membrane surface can interact with the membrane’s Cu ions to create more hydroxyl radicals. On the other hand, the coating of Ag may mix with the water’s electrons to generate OH- and further form the hydroxyl radicals reacting with H_2_O. These hydroxyl radicals on the CC@Cu:TiO_2_@Ag_Y_ membranes will improve membrane hydrophilicity, eventually enhancing membrane oleophobicity. In our case, Cu doping and Ag coating can change the U-OCA. However, the U-OCA of the CC@Cu:TiO_2_@Ag_Y_ membranes decreases when the value of Y increases to 0.4 due to the blocked microstructure by superfluous Ag. Figure 6 depicts the hydrophilicity of the CC@Cu:TiO_2_@Ag_0.3_ membrane in air, the oleophobicity of the membrane in water and its dynamic oleophobic diagram. According to this figure, the water contact angle (WCA) of the CC@Cu:TiO_2_@Ag_0.3_ membrane in the air is 0°, indicating that water can easily pass through the membrane. That is to say, the membrane has the superhydrophilicity. In addition, an oil droplet can potentially not leave any residue on superoleophobic surfaces even when the oil droplet is deformed and has contacted the membrane surface multiple times, indicating the low oil adhesion of the membranes. The membrane with the lowest oil adhesion is shown by an external force. All of the foregoing investigations show that the constructed CC@Cu:TiO_2_@Ag_0.3_ membrane has good antifouling performance as well as superhydrophilicity/underwater superoleophobicity, implying that it has a bright future in oil–water separation.

**Figure 7:**
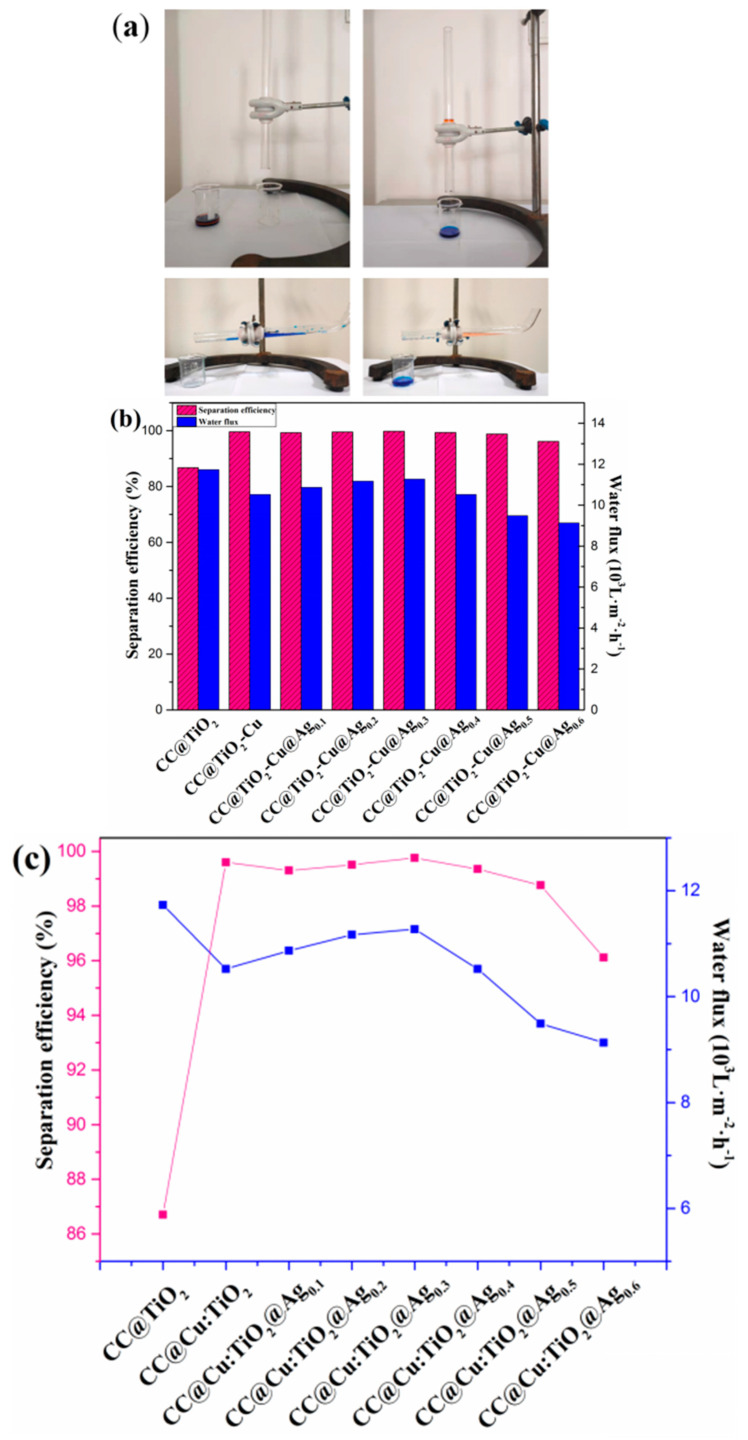
(**a**) The oil–water separation devices, (**b**,**c**) the separation efficiency and the water flux of n-hexane/water mixtures for CC@TiO_2_, CC@Cu:TiO_2_ and CC@Cu:TiO_2_@ Ag_Y_ (Y = 0.1, 0.2, 0.3, 0.4, 0.5 and 0.6) membranes.

**Figure 8:**
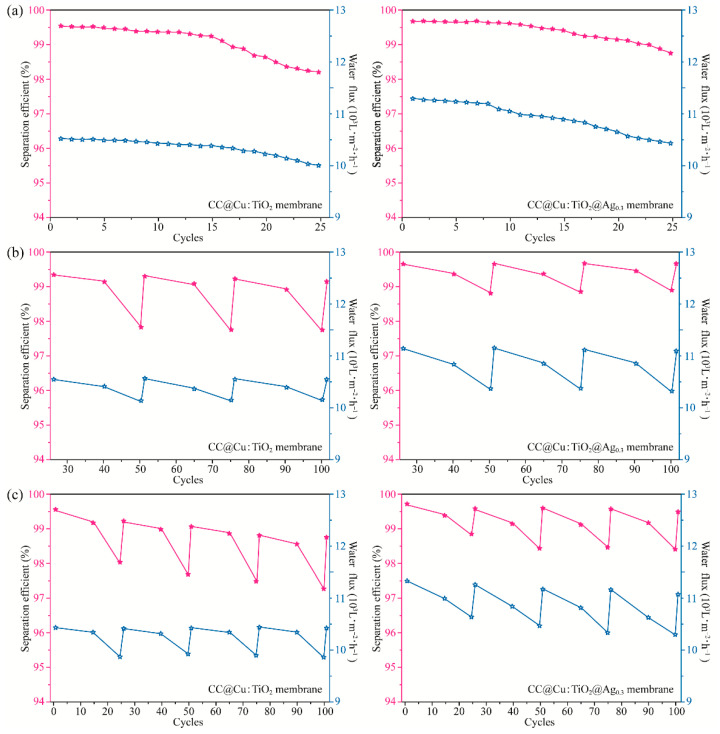
(**a**) The separation efficiency and water flux of the CC@Cu:TiO_2_ and CC@Cu:TiO_2_ @Ag_0.3_ membranes after 25 cycles, (**b**) the separation efficiency and water flux of CC@Cu:TiO_2_ and CC@Cu:TiO_2_ @Ag_0.3_ membranes in 75 cycles (irradiation time of 30 min), (**c**) the separation efficiency and water flux of CC@Cu:TiO_2_ and CC@Cu:TiO_2_ @Ag_0.3_ membranes after 100 cycles (irradiation time of 15 min). The n-hexane/water mixture was used in the experiment.

**Figure 9:**
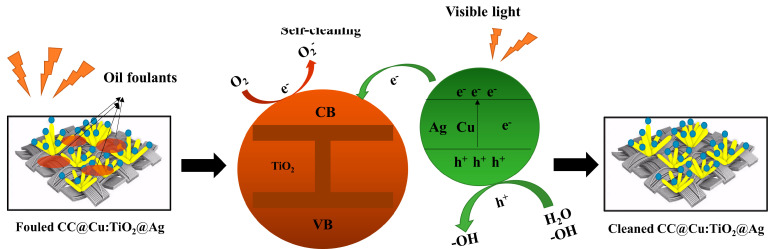
The self-cleaning mechanism of the CC@Cu:TiO_2_@Ag membrane.

The oil–water separation test of the membranes is carried out to confirm the initial analysis further and look into the effectiveness of separation and self-cleaning. Figure 7 shows the oil–water separation equipment (a). The tested membranes are clamped onto the two PTFE flange ends with an inner diameter of 18 mm, as illustrated in the oil–water separation device, and the two quartz tubes with a diameter of 15 mm are then placed into the end of the PTFE flange. The produced membranes should be moistened before the separation experiment, and then the mixture of 5 mL of oil and 5 mL of water is poured into the separation device. Briefly, 5 mL of deionized water is colored blue with methylene blue (MB), and 5 mL of oil is colored red with Sudan III for simple observation. When the oil–water mixture is poured into the device, the water barrier generated by the wet membrane prevents the oil from passing through the CC-based membrane. Still, the water can move through the membrane quickly using gravity alone. Figure 7b,c shows that the separation efficiency of the CC@TiO_2_ membrane without doping and coating is 86.71%, and that of the CC@Cu:TiO_2_ membrane is boosted to 99.54% due to the larger U-OCA mentioned above, indicating that Cu doping plays a crucial role in oil–water separation efficiency. Moreover, the separation efficiency of the doped and coated membranes of CC@Cu:TiO_2_@Ag_Y_ first increases and then decreases as the Ag content increases, and the value reaches 99.76% when the Ag content is fixed at 0.003 mol/L. The water flux of the prepared membranes gradually decreases due to the block gap of the CC when incorporating the Cu and Ag ions. Based on the above analyses, the CC@Cu:TiO_2_@Ag_0.3_ membrane is thought to have the best oil–water separation. In order to further research the significance of the role of Ag, the long cycle oil–water separation experiment for the CC@Cu:TiO_2_ and CC@Cu:TiO_2_@Ag_0.3_ membranes is performed as shown in Figure 8. In this experiment, the membranes are tested for 100 cycles, and every 25 cycles is set as a big cycle. After a big cycle, the membranes are put into 30 mL of deionized water and irradiated with visible light for 15 min and 30 min, respectively. For the first big cycle shown in Figure 8a, the separation efficiency and the water flux for the two membranes both have minor drops from 99.54% to 98.20%, 10524 Lm^−2^h^−1^ to 10010 Lm^−2^h^−1^ (CC@Cu:TiO_2_), 99.76% to 98.77% and 11274 Lm^−2^h^−1^ to 10422 Lm^−2^h^−1^ (CC@Cu:TiO_2_@Ag_0.3_), respectively. The minor drop may be caused by the oil residue attached to the membrane after multiple oil–water separation tests. Before the next big cycle, the membrane is given the self-cleaning treatment at the same irradiation time of 30 min as mentioned above. It is found that the separation efficiency of the CC@Cu:TiO_2_ and CC@Cu:TiO_2_@Ag_0.3_ can be restored to 99.32% and 99.65%, and the water flux can be restored to 10530 Lm^−2^h^−1^ and 11127 Lm^−2^h^−1^, respectively. The above values after 100 cycles can even reach 99.12% and 10522 Lm^−2^h^−1^ (CC@Cu:TiO_2_), and 99.65% and 11074 Lm^−2^h^−1^ (CC@Cu:TiO_2_@Ag_0.3_), demonstrating the superior stability and repeatability of the membranes (Figure 8b). Importantly, the above performance of the CC@Cu:TiO_2_@Ag_0.3_ membrane after a short irradiation time of 15 min is also much better than that of the CC@Cu:TiO_2_ membrane under a longer irradiation time of 30 min (Figure 8c). It indicates that the CC@Cu:TiO_2_@Ag_0.3_ membrane has the best self-cleaning performance that can be restored in a short time due to the Ag coating. Two reasons can account for it, and the self-cleaning mechanism can be seen in Figure 9. For one reason, the membrane’s visible light response can be enhanced by incorporating Ag due to the surface plasmon resonance (SPR) effect [56]. Another reason is that the catalytic degradation performance of the membranes can also be improved to some extent due to the adjusted bandgap structure. In the self-cleaning process, the photo-induced photons can be absorbed by the trap sites of Ag, and subsequently, some electrons are produced under the influence of visible light. The electrons from SPR are partially injected into the conduction band of TiO_2_ because of the higher Fermi energy level of Ag. The growth of the conjugate structure also enables certain electrons in the TiO_2_ conduction band to transfer quickly onto the CC surface, enhancing electron–hole separation. Moreover, the O_2_ molecules on the TiO_2_ surface can be converted into O_2_^−^ by capturing the electrons [57]. Therefore, the degradation ability of pollutants will be effectively enhanced by a certain amount of Ag coating for the CC@Cu:TiO_2_@Ag_0.3_ membrane. Thus, the CC@Cu:TiO_2_@Ag_0.3_ membrane has excellent self-cleaning performance, and the Ag coating plays a main role in it.

**Figure 10:**
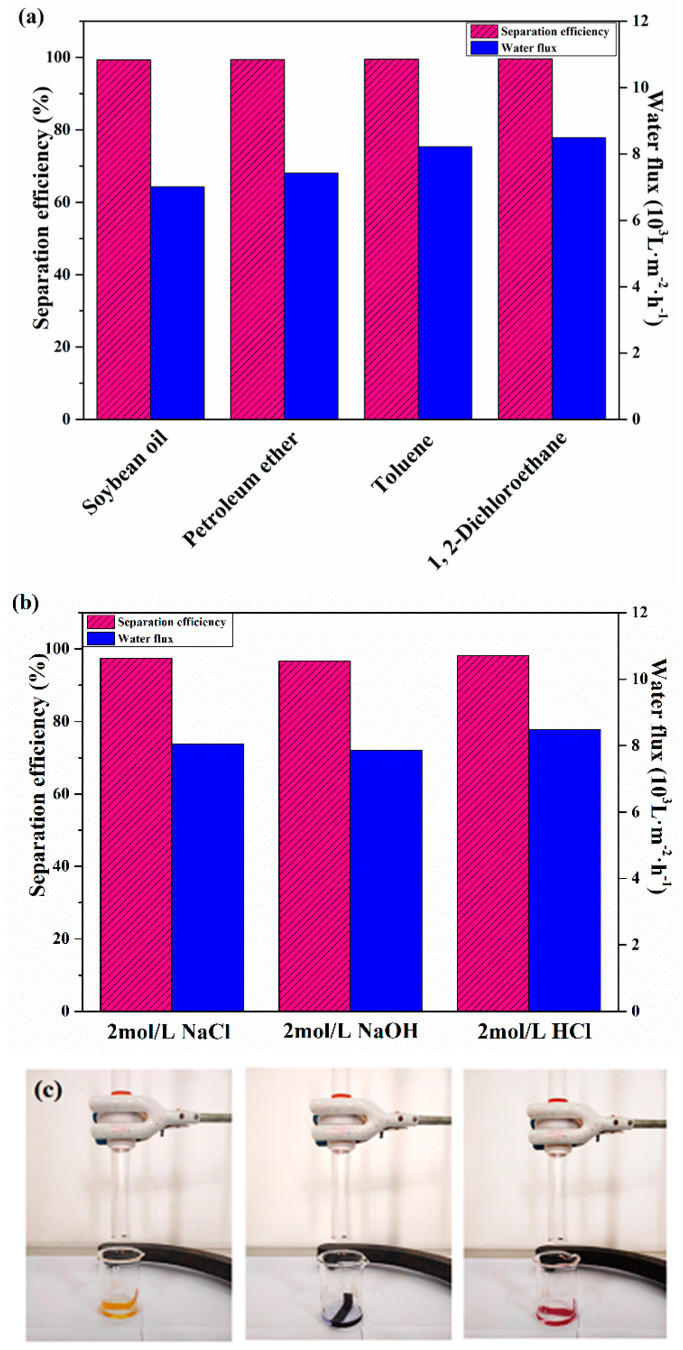
(**a**) The separation efficiency and the water flux of various oils (soybean oil, petroleum ether, toluene and 1, 2- dichloroethane) for the CC@Cu:TiO_2_@Ag_0.3_ membrane, (**b**) The separation efficiency and water flux of acid–base salt for CC@Cu:TiO_2_@Ag_0.3_ membrane and (**c**) their separation pictures.

In order to make the separation environment more suitable for polluted wastewater, the oil–water separation performance of the CC@Cu:TiO_2_@Ag_0.3_ membrane has been tested by using various oils such as toluene, petroleum ether, soybean oil and 1, 2-dichloroethane. Soybean oil, petroleum ether, toluene and 1, 2-dichloroethane have separation efficiencies and water fluxes of 99.35% and 7020 Lm^−2^h^−1^, 99.38% and 7429 Lm^−2^h^−1^, 99.51% and 8221 Lm^−2^h^−1^ and 99.6% and 8502 Lm^−2^h^−1^, respectively, as illustrated in Figure 10a. The separation efficiency of various oil–water mixtures with the CC@Cu:TiO_2_@Ag_0.3_ membrane may vary. The separation efficiencies are all over 99%, indicating that the prepared membrane with the higher oil–water performance perhaps can be used in various oil–water mixture treatments. Figure 10b,c shows the viability tests of the CC@Cu:TiO_2_@Ag_0.3_ membrane in acidic, alkaline and high-salt environments. The separation efficiency of the CC@Cu:TiO_2_@Ag_0.3_ membrane in the above environments is above 96% in our case, illustrating that the CC@Cu:TiO_2_@Ag_0.3_ membrane exhibits the ability to separate an oil–water mixture under harsh acidic, alkaline and high-salt environments of with 2 mol/L.

**Figure 11:**
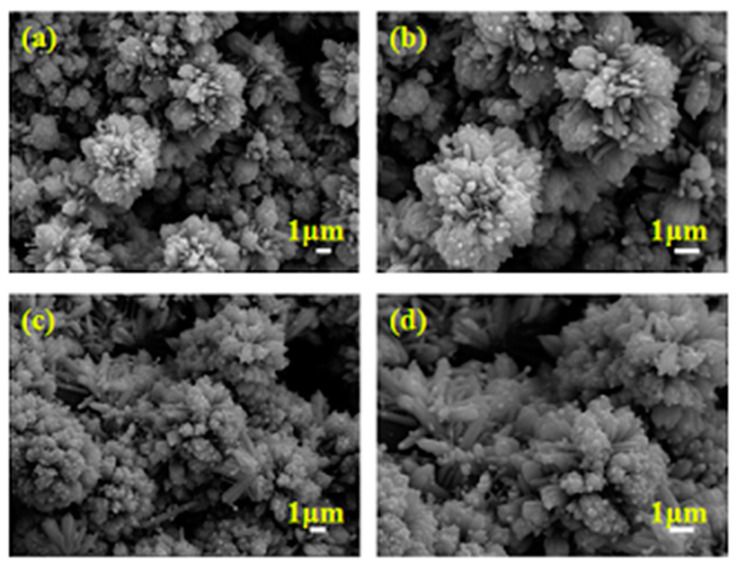
(**a,b**) SEM images of CC@Cu:TiO_2_@Ag_0.3_ membrane before oil–water separation at 5000 magnification and 8000 magnification, (**c**,**d**) SEM images of CC@Cu:TiO_2_@Ag_0.3_ membrane after oil–water separation at 5000 magnification and 8000 magnification.

**Figure 12:**
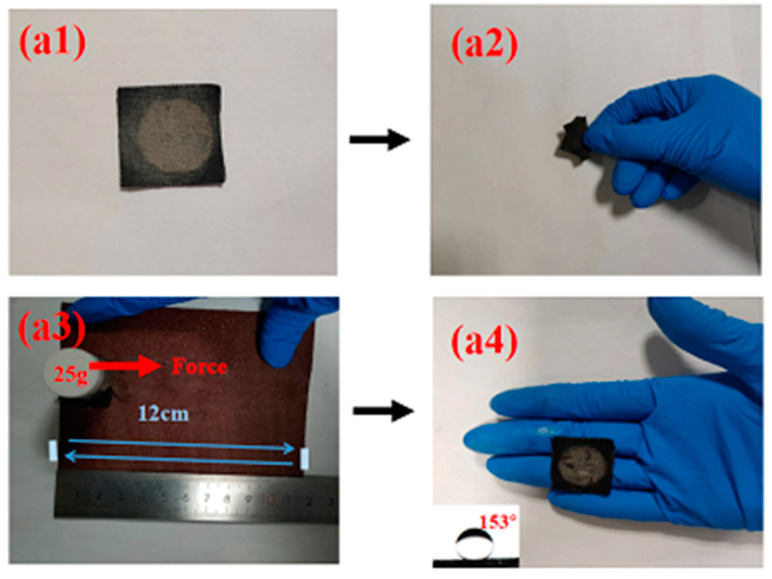
(**a1**,**a2**) The flexibility test and (**a3**,**a4**) durability test of the CC@Cu:TiO_2_@Ag_0.3_ membrane.

Next, the stability and repeatability tests of the CC@Cu:TiO_2_@Ag_0.3_ membrane have been conducted. Figure 11 shows the morphology of the CC@Cu:TiO_2_@Ag_0.3_ membrane before and after all of the oil–water separation experiments mentioned above. Compared with the SEM image before oil–water separation, the overall appearance of the membrane after oil–water separation is unchanged. The original CC is still covered by the Cu:TiO_2_@Ag structure and a chrysanthemum-like structure can be seen on the original basis. It shows that the prepared membrane not only has an extremely high separation efficiency but also has excellent stability and repeatability. Folding and abrasion tests have also been carried out to prove the mechanical strength of the CC@Cu:TiO_2_@Ag_0.3_ membrane, shown in Figure 12. It can be observed that the separation efficiency can still reach 99.6% after the membrane is folded 500 times. Notably, when the CC@Cu:TiO_2_@Ag_0.3_ membrane, attached underneath a weight of 25 g using sandpaper as the base, is pulled for 12 cm and then back to its starting point (every 24 cm named as one cycle), the U-OCA of this membrane is still 153° after abrading for five cycles. It demonstrates that the engineered CC-based membrane has high durability.

**Figure 13:**
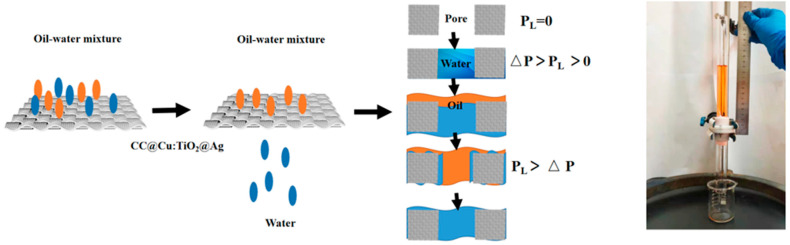
The oil–water separation mechanism of CC@Cu:TiO_2_@Ag membrane and the intrusion pressure of oil.

The oil–water separation mechanism is presented to illustrate the separation process. In this experiment, the membrane is first wetted by the water and then 1, 2-dichloroethane (dyed by Sudan III) is poured into the oil–water separation device until the oil droplets penetrate the membrane completely. The intrusion pressure (P_I_) is a pressure threshold for the maximum height of an oil column that the membrane can withstand. Through the formula of P_I_ = ρgh_max_ (ρ means the density of 1, 2-dichloroethane, and g means the gravitational acceleration) [55], the intrusion pressure is measured on the membrane surface at the maximum allowable liquid height. The maximum liquid column of h_max_ for the membrane is roughly 13.5 cm, as seen in Figure 13; hence, P_I_ is computed to be 1.663 kPa. When the liquid pressure is less than 1.663 kPa, the oil cannot pass through the membrane.

## 4. Conclusions

These hydrothermal and photodeposition methods are used to create a novel engineered CC-based membrane. Cu doping and Ag coating have been found to be beneficial for the excellent oil-separation efficiency and self-cleaning ability of membranes with superhydrophilicity/underwater superoleophobicity due to increased hydroxyl radical generation and enhanced visible light responsiveness. The CC@Cu:TiO_2_@Ag_0.3_ membrane shows the best performance when the underwater oil contact angle is 156° and the separation efficiency is up to 99.76%. Additionally, the membrane’s ability to separate oil from water can be almost recovered under visible light driven for 15 min, indicating that the membrane has excellent self-cleaning ability. Importantly, the membrane can be worked with high separation efficiency in various oils and corrosive environments and even has good durability and wear resistance. The advantages of the simple preparation method and the excellent performance mentioned above indicate that the membranes will have a wide range of practical wastewater treatment applications.

## Data Availability

The data presented in this study are available in the article.
